# PD-L1 Is an Independent Prognostic Marker in Middle Eastern PTC and Its Expression Is Upregulated by *BRAFV600E* Mutation

**DOI:** 10.3390/cancers13030555

**Published:** 2021-02-01

**Authors:** Abdul K. Siraj, Sandeep Kumar Parvathareddy, Poyil Pratheeshkumar, Sasidharan Padmaja Divya, Saif S. Al-Sobhi, Fouad Al-Dayel, Khawla S. Al-Kuraya

**Affiliations:** 1 Human Cancer Genomic Research, Research Center, King Faisal Specialist Hospital and Research Center, P.O. Box 3354, Riyadh 11211, Saudi Arabia; asiraj@kfshrc.edu.sa (A.K.S.); psandeepkumar@kfshrc.edu.sa (S.K.P.); ppoyil@kfshrc.edu.sa (P.P.); pdivya@kfshrc.edu.sa (S.P.D.); 2 Department of Surgery, King Faisal Specialist Hospital and Research Center, P.O. Box 3354, Riyadh 11211, Saudi Arabia; sobhi@kfshrc.edu.sa; 3 Department of Pathology, King Faisal Specialist Hospital and Research Centre, P.O. Box 3354, Riyadh 11211, Saudi Arabia; dayelf@kfshrc.edu.sa

**Keywords:** PD-L1, *BRAFV600E* mutation, papillary thyroid cancer, recurrence-free survival, cell growth, vemurafenib

## Abstract

**Simple Summary:**

This study was conducted to investigate the prognostic significance of programmed death-ligand 1 (PD-L1) expression in a large cohort of Middle Eastern papillary thyroid carcinoma (PTC) patients and to explore the correlation of PD-L1 and *BRAFV600E* mutations in PTC tumors and cell lines. We found PD-L1 over-expression in PTC patients and it was significantly associated with aggressive clinico-pathological parameters and *BRAF* mutation. PTC patients with co-existing PD-L1 over-expression and *BRAF* mutation had a poor disease-free survival. In vitro studies showed that BRAF inhibition induces PD-L1 expression in *BRAF*-mutated PTC cell lines via mitogen-activated protein kinase kinase/extracellular-signal-regulated kinase (MEK/ERK) pathway activation. Silencing of PD-L1 in *BRAF*-mutated cell lines significantly attenuated cell growth. Our data suggest that PD-L1 could represent a useful prognostic marker for risk stratification in Middle Eastern PTC and that a programmed cell death protein 1 (PD-1)/PD-L1 inhibitor could be a potential therapeutic option for aggressive PTC cancers, such as the tall cell variant, *BRAF* mutation-positive patients that are unresponsive to standard PTC treatment.

**Abstract:**

PD-L1 inhibition is a promising therapeutic target whose efficacy has been demonstrated in several cancers. Immunohistochemistry was performed to assess PD-L1 protein expression in PTC. We further conducted in vitro analysis to investigate the role of PD-L1 in regulating *BRAFV600E* in PTC cell lines. PD-L1 over-expression was noted in 32.4% (473/1458) of cases and significantly associated with aggressive clinico-pathological parameters. Importantly, PD-L1 was found to be an independent poorer prognostic marker. We also found PD-L1 to be significantly associated with *BRAF* mutation and patients with co-existing PD-L1 over-expression and *BRAF* mutation had a poor disease-free survival compared to patients with *BRAF* mutation alone. In vitro analysis showed high expression of PD-L1 in *BRAF*-mutated PTC cell lines compared to a *BRAF* wild-type cell line. Inhibition of BRAF using vemurafenib induced PD-L1 expression in *BRAF*-mutated cell lines without affecting cell growth. Knockdown of PD-L1 in *BRAF*-mutated cell lines significantly decreased the cell growth and induced apoptosis. Our data suggest that PD-L1 might represent a useful prognostic marker in Middle Eastern PTC and PD-L1 inhibition could be a potential therapeutic option for aggressive PTC cancers, such as the tall cell variant, *BRAF* mutation-positive patients that are unresponsive to standard treatment.

## 1. Introduction

Papillary thyroid carcinoma (PTC) is the commonest among all thyroid carcinomas [1,2]. Although PTCs are indolent, successfully curable and have an overall good prognosis, however, 20% of PTCs show recurrence and about 5% manifest with distant metastasis and may become resistant to radioactive iodine therapy [3,4,5]. Therefore, identifying new molecular targets that could predict prognosis is essential to overcome adverse outcomes in PTC patients.

Recently, one of the potential targets that has been under close scrutiny is programmed cell death ligand 1 (PD-L1) [6,7]. PD-L1 is a key immune regulatory molecule that interacts with programmed cell death protein (PD-1) to suppress T cell immune responses that help the tumor cells to escape the immune system [8,9]. Blockade of the PD-1/PD-L1 pathway with monoclonal antibodies is a promising therapeutic strategy that shows strong clinical benefits in multiple malignancies [10,11,12,13]. Despite PD-L1 protein expression being used as a predictive marker of therapeutic response to PD-L1 inhibitors in several cancers [14,15,16,17], there are many cancers that fail to respond to anti-PD-1/PD-L1 therapies. A recent clinical trial (Phase 1b KEYNOTE-028) in 22 advanced PTCs and follicular thyroid cancers evaluated the safety and antitumor activity of pembrolizumab as monotherapy. Only two patients showed a partial response (overall response rate = 9%) [18]. This might be explained by the ability of PD-L1 to regulate tumor cells in an immune-independent manner [19,20]. Indeed, several reports have shown that PD-L1 could be involved in regulation of signaling pathways [21,22,23,24].

PTC is a predominantly MAP kinase signaling pathway-driven cancer [25]. The *BRAFV600E* mutations represent the most common genetic alteration in PTC and they has been shown to predict PTC aggressiveness and patient prognosis [3,26]. Increased PD-L1 expression has been shown previously to be associated with *BRAFV600E* point mutation in several cancers including thyroid cancer [27,28,29]. Moreover, a recent report has demonstrated that *BRAFV600E* mutation can upregulate PD-L1 expression, which further supports the non-immune function of PD-L1 [30].

The level of PD-L1 expression in PTC and overall prognosis have shown conflicting data [31,32,33]. However, information about PD-L1 expression in PTCs from people of Middle Eastern ethnicity (where PTC prevalence is very high) has never been explored before. Therefore, we conducted a comprehensive analysis to evaluate the clinico-pathological and prognostic significance of PD-L1 expression in a large cohort of Middle Eastern PTC patients. Given the significant association of PD-L1 and *BRAFV600E* mutation in our cohort, we explored whether PD-L1 is regulated by *BRAFV600E* using PTC cell lines.

## 2. Results

### 2.1. Programmed Cell Death Ligand 1 (PD-L1) Expression in Papillary Thyroid Carcinoma (PTC) and Its Clinico-Pathological Associations

**Figure 1:**
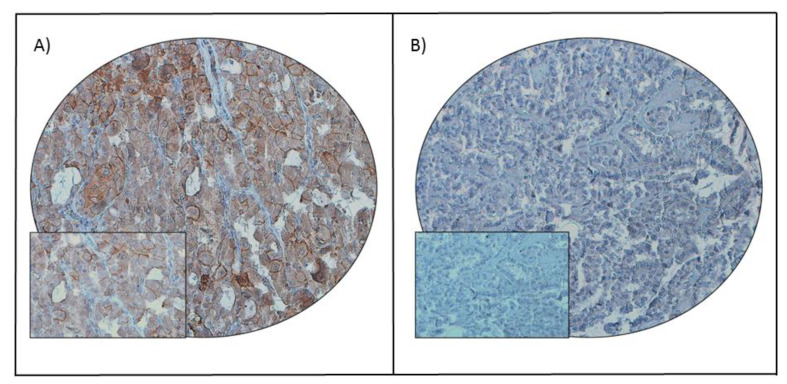
PD-L1 immunohistochemical staining in papillary thyroid carcinoma (PTC) tissue microarray (TMA). Representative examples of tumors showing (**A**) high expression and (**B**) low expression (right panel) of PD-L1. A 20 ×/0.70 objective on an Olympus BX 51 microscope (Olympus America Inc., Center Valley, PA, USA) with the inset showing a 40 × 0.85 aperture magnified view of the same TMA spot.

**Figure 2:**
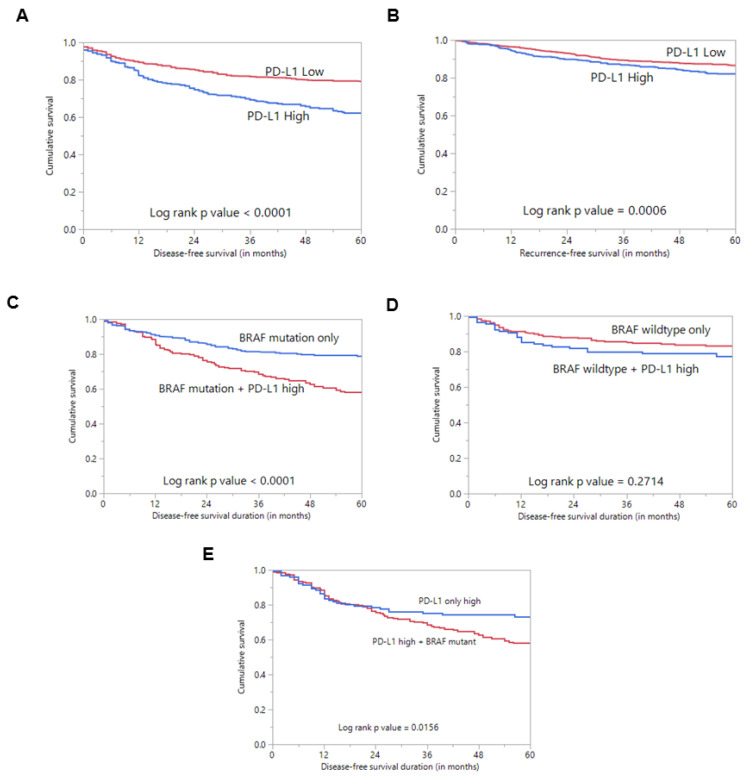
Survival analysis of PD-L1 protein expression. (**A**) Kaplan–Meier survival plot showing statistically significant poor disease-free survival in PD-L1 high-expression cases compared to PD-L1 low expression (*p* < 0.0001). (**B**) Kaplan–Meier survival plot showing statistically significant poor recurrence-free survival in PD-L1 high-expression cases compared to PD-L1 low expression (*p* = 0.0006). (**C**) Kaplan–Meier survival plot showing statistically significant poor disease-free survival in cases with co-existing PD-L1 expression and *BRAF* mutation compared to cases with *BRAF* mutation alone (*p* < 0.0001). (**D**) Kaplan–Meier survival plot shows no statistically significant difference in disease-free survival in cases with co-existing PD-L1 expression and wildtype *BRAF* compared to cases with wildtype *BRAF* alone (*p* = 0.2714). (**E**) Kaplan–Meier survival plot showing statistically significant poor disease-free survival in cases with co-existing PD-L1 expression and *BRAF* mutation compared to cases with PD-L1 expression alone (*p* = 0.0156).

PD-L1 protein expression was assessed immunohistochemically in 1512 PTC samples. However, immunohistochemistry data were interpretable in 1458 samples and hence were included for further analysis. PD-L1 over-expression was noted in 32.4% (473/1458) of cases (Table 1; Figure 1). A significant association was noted between PD-L1 over-expression and aggressive clinico-pathological characteristics such as tall cell variant (*p* < 0.0001), extrathyroidal extension (*p* = 0.0203) and lymph node metastasis (*p* = 0.0466) (Table 1). Importantly, we also found a significant association between PD-L1 over-expression and poor disease-free survival (DFS; *p* < 0.0001), as well as poor recurrence-free survival (RFS; *p* = 0.0006) (Table 1; Figure 2A,B), but not overall survival (*p* = 0.0921). On multivariate analysis, PD-L1 was found to be an independent predictor of DFS (HR = 2.16; 95% CI = 1.73–2.72; *p* < 0.0001) and RFS (HR = 1.59; 95% CI = 1.22–2.05; *p* = 0.0005) (Table 2).

Interestingly, we found an association between PD-L1 over-expression and *BRAF* mutation (*p* = 0.0183) (Table 1). Since *BRAFV600E* mutation is known to play a role in the pathogenesis and prognosis of PTC, we sought to determine whether the prognostic associations were independently driven by PDL1 expression rather than co-existing *BRAFV600E* mutation. Using Kaplan–Meier analysis, we found that cases with co-existing PD-L1 over-expression and *BRAF* mutation had a significantly worse DFS compared to cases with *BRAF* mutation alone (*p* < 0.0001) (Figure 2C).

### 2.2. BRAF Mutation and Its Association with PD-L1 in PTC In Vitro

**Figure 3:**
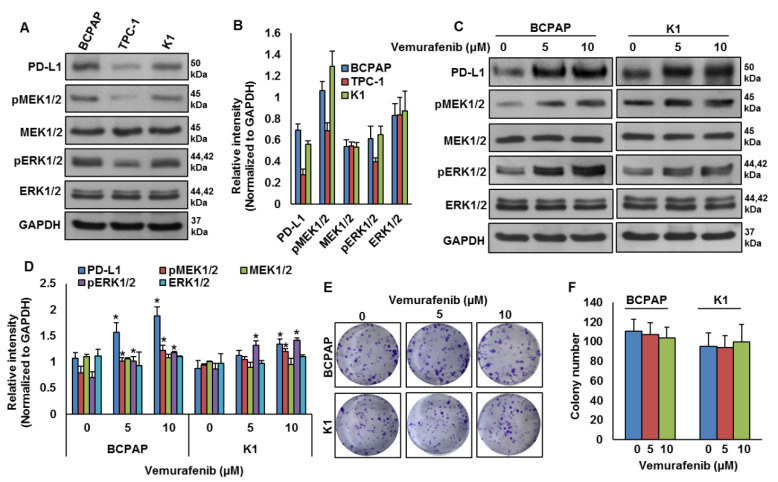
*BRAF* mutation and its association with PD-L1 in PTC in vitro. (**A**,**B**) Basal expression of PD-L1 in PTC cell lines. Proteins were isolated from three PTC cell lines and immuno-blotted with antibodies against PD-L1, pMEK1/2, MEK1/2, pERK1/2, ERK1/2 and GAPDH. Western blots were quantified and data are presented as mean ± SD of three independent experiments (*n* = 3). (**C**,**D**) BRAF inhibition increases PD-L1 expression. *BRAF*-mutated PTC cell lines were treated with indicated doses of vemurafenib for 48 h. After cell lysis, equal amounts of proteins were separated by SDS-PAGE, transferred to immobilon membrane, and immuno-blotted with antibodies against PD-L1, pMEK1/2, MEK1/2, pERK1/2, ERK1/2 and GAPDH as indicated. Western blots were quantified and data are presented as mean ± SD of three independent experiments (*n* = 3). * Indicates a statistically significant difference compared to control with *p* < 0.05. (**E**,**F**) BRAF inhibition on cell growth. *BRAF*-mutated PTC cell lines were treated with indicated doses of vemurafenib for 48 h. Cells (5 × 10^2^) were then re-seeded into a 6-well plate, and grown for an additional 6–8 days, then stained with crystal violet and colonies were counted. Data presented in the bar graphs are the mean ± SD of three independent experiments (*n* = 3) which were repeated at least two times with the same results. * Indicates a statistically significant difference compared to control with *p* < 0.05.

Our clinical data showed a significant association between PD-L1 over-expression and *BRAF* mutation. To test this association in vitro, we analyzed the basal expression of PD-L1 in PTC cell lines by immuno-blotting. We found high expression of PD-L1 in *BRAF*-mutated PTC cell lines (BCPAP and K1) compared to a wildtype *BRAF* PTC cell line (TPC-1) (Figure 3A,B). We also found increased expression of pMEK1/2 and pERK1/2 in *BRAF*-mutated cell lines compared to a wildtype cell line (Figure 3A,B). Next, we inhibited BRAF using vemurafenib and analyzed the expression of PD-L1, pMEK1/2 and pERK1/2 in *BRAF*-mutated cell lines. As shown in Figure 3C,D, inhibition of BRAF induced PD-L1, pMEK1/2 and pERK1/2 expressions in *BRAF*-mutated cell lines. However, vemurafenib treatment did not affect the colony-forming ability of PTC cells, as shown by clonogenicity assay (Figure 3E,F).

### 2.3. Mitogen-Activated Protein Kinase Kinase (MEK) Inhibition Decreases PD-L1 Expression

**Figure 4:**
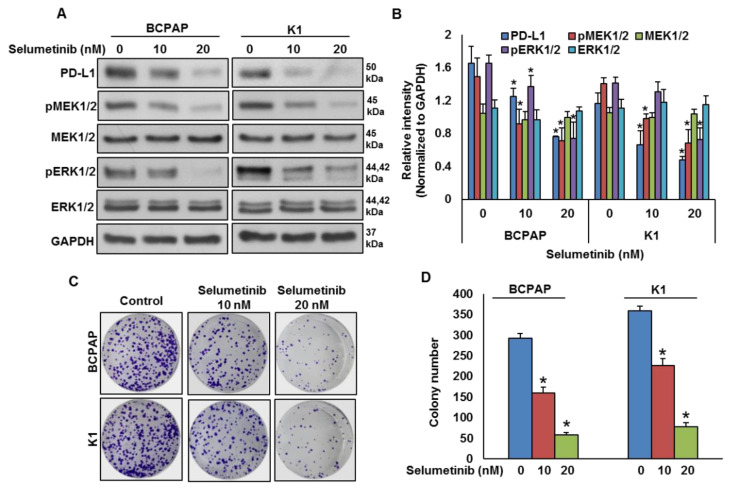
MEK inhibition downregulates PD-L1 expression and decreases cell growth. (**A**,**B**) MEK inhibition downregulates PD-L1 expression. *BRAF*-mutated cells were treated with indicated doses of selumetinib for 48 h. After cell lysis, equal amounts of proteins were separated by SDS-PAGE, transferred to immobilon membrane, and immuno-blotted with antibodies against PD-L1, pMEK1/2, MEK1/2, pERK1/2, ERK1/2 and GAPDH as indicated. Western blots were quantified and data are presented as mean ± SD of three independent experiments (*n* = 3). * Indicates a statistically significant difference compared to control with *p* < 0.05. (**C**,**D**) MEK inhibition decreases cell growth. *BRAF*-mutated PTC cell lines were treated with indicated doses of selumetinib for 48 h. Cells (5 × 10^2^) were then re-seeded into a 6-well plate, and grown for an additional 6–8 days, then stained with crystal violet and colonies were counted. Data presented in the bar graphs are the mean ± SD of three independent experiments (*n* = 3) which were repeated at least two times with the same results. * Indicates a statistically significant difference compared to control with *p* < 0.05.

To test the effect of MEK inhibition on PD-L1 expression, *BRAF*-mutated cell lines were treated with a pharmacologic inhibitor for MEK, selumetinib, for 48 h and the expressions of PD-L1, pMEK1/2 and pERK1/2 were analyzed by immuno-blotting. As shown in Figure 4A,B, inhibition of MEK by selumetinib prominently downregulated the expressions of PD-L1, pMEK1/2 and pERK1/2 in *BRAF*-mutated cell lines in a dose-dependent manner. Furthermore, clonogenic ability was significantly reduced post-selumetinib treatment as compared to untreated control (Figure 4C,D).

### 2.4. Downregulation of PD-L1 Decreases Cell Growth of BRAF-Mutated Cell Lines

**Figure 5:**
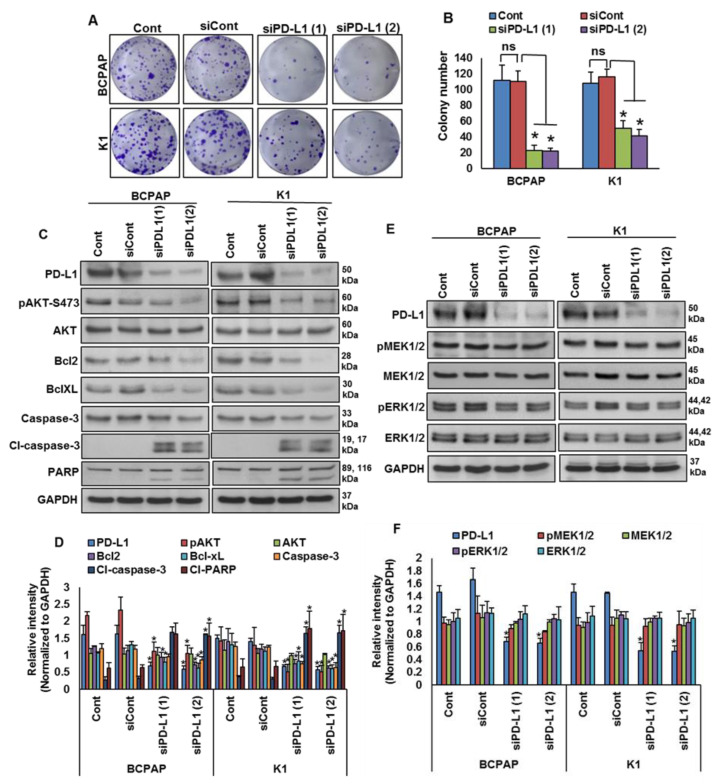
Silencing of PD-L1 decreases cell growth of *BRAF*-mutated cell lines. (**A**,**B**) Knockdown of PD-L1 decreases clonogenicity. *BRAF*-mutated PTC cells were transfected with scrambled siRNA and two different PD-L1 siRNAs (50 nM). Forty-eight hours post-transfection, cells (5 × 10^2^) were re-seeded into a 6-well plate, and grown for an additional 6–8 days, then stained with crystal violet and colonies were counted. Data presented in the bar graphs are the mean ± SD of three independent experiments (*n* = 3), which were repeated at least two times with the same results. * Indicates a statistically significant difference compared to siRNA control with *p* < 0.05. (**C**,**D**) Knockdown of PD-L1 decreases AKT phosphorylation and downregulates the expression of anti-apoptotic proteins and induces the cleavage of caspase-3 and PARP. *BRAF*-mutated PTC cells were transfected with scrambled siRNA and two different PD-L1 siRNAs (50 nM). Forty-eight hours post-transfection, cells were lysed and equal amounts of proteins were separated and immuno-blotted with antibodies against PD-L1, pAKT, AKT, Bcl-2, Bcl-xL, caspase-3, cleaved caspase-3, PARP and GAPDH as indicated. Western blots were quantified and data are presented as mean ± SD of three independent experiments (*n* = 3). * Indicates a statistically significant difference compared to siControl with *p* < 0.05. (**E**,**F**) Knockdown of PD-L1 caused no effect on MEK/ERK activation. *BRAF*-mutated PTC cells were transfected with scrambled siRNA and two different PD-L1 siRNAs (50 nM). Forty-eight hours post-transfection, cells were lysed and equal amounts of proteins were separated and immuno-blotted with antibodies against PD-L1, pMEK1/2, MEK1/2, pERK1/2, ERK1/2 and GAPDH. Western blots were quantified and data are presented as mean ± SD of three independent experiments (*n* = 3). * Indicates a statistically significant difference compared to siControl with *p* < 0.05.

We showed that PD-L1 over-expression was significantly associated with *BRAF* mutation and poor survival in PTC patient samples. Therefore, we sought to determine whether targeting PD-L1 expression would be a viable therapeutic strategy to inhibit growth of *BRAF*-mutated cells. We knocked down PD-L1 in *BRAF*-mutated cells using specific siRNA and analyzed the cell growth by a clonogenicity assay. Knockdown of PDL1 using two different siRNA sequences significantly decreased the clonogenic ability of PTC cells after 48 h of transfection (Figure 5A,B). In addition, silencing of PD-L1 decreased AKT-Ser (473) phosphorylation, and downregulated anti-apoptotic proteins bcl2 and bcl-xL, as well as induced cleavage of caspase-3 and PARP, in *BRAF*-mutated cell lines (Figure 5C,D). However, knockdown of PD-L1 did not change the expressions of pMEK1/2 and pERK1/2, showing that PD-L1 functions downstream of the MEK/ERK signaling cascade (Figure 5E,F).

## 3. Discussion

PTC accounts for more than 80% of all thyroid cancers and is typically associated with a favorable prognosis [34,35,36]. Aside from radioactive therapy, which is the standard care, there are limited therapeutic options available for the small percentage of patients who have aggressive PTC and eventually develop resistance to radioactive iodine treatment [37,38]. Recently, treatment with PD-1/PD-L1 inhibitors has demonstrated a therapeutic effect in several aggressive tumors [11,12,13]. PD-L1 expression has been used to identify patients who are likely to respond to anti-PD-L1 therapies [39,40]. Furthermore, PD-L1 expression has emerged as a potential prognostic marker in several solid tumors including thyroid cancer [31,41,42,43,44]. In this study, we evaluated the association between PD-L1 over-expression and clinico-pathological markers as well as survival in patients with PTC. Analyzing more than 1400 Middle Eastern PTC patients from a single institute demonstrated the prognostic value of PD-L1 expression in these patients.

We found a statically significant correlation between higher levels of PD-L1 expression and lymph node metastasis, extrathyroidal extension, tall cell variant, DFS and RFS. Several previous studies evaluated the diagnostic and prognostic value of PD-L1 expressions in thyroid cancer without reaching consensus [31,32,33,45]. These conflicting data could be attributed to several factors, including sample size, antibody used, cut-off values applied and ethnicity of the patients. Analogous to our findings, a study of 185 PTCs from Canada showed a significantly shorter median DFS in PD-L1-positive tumors compared to PD-L1-negative tumors [33]. Similarly, another study on 260 PTC cases showed a significantly worse RFS in PD-L1-positive tumors in multivariate Cox regression analysis [31].

Interestingly, we found a significant positive association between PD-L1 expression and the presence of *BRAFV600E* mutations, which is known to play a major role in PTC pathogenesis and aggressiveness. This led us to question whether the prognostic associations were independently driven by PD-L1 expression rather than co-existing *BRAFV600E* mutation because >50% of PD-L1-positive PTC samples had co-existing *BRAFV600E* mutations. Specifically, we compared the DFS in cases with coexisting PD-L1 expression and *BRAFV600E* mutation to those with *BRAFV600E* mutation alone and found the former to be associated with significantly poorer DFS (*p* < 0.0001). This finding could support a previous report highlighting the ability of *BRAFV600E* signaling to modulate the immune response [29]. Interestingly, a recent study in colorectal cancer (CRC) has shown that *BRAFV600E* can transcriptionally upregulate PD-L1 expression and enhance apoptosis which might indicate an intrinsic non-immune function of PD-L1 [30].

Thus, we determined whether *BRAFV600E* can regulate PD-L1 expression in human PTC cell lines. Our in vitro studies confirmed the association of high PD-L1 expression in the PTC-*BRAF*-mutated cell lines. Vemurafenib treatment of the *BRAF*-mutated cell lines induces PD-L1 expression, as shown by western blotting, without affecting cell viability. The mitogen activated protein kinase (MAPK) pathway has been shown to regulate PD-L1 expression in various cancers [46,47,48]. In PTC cell lines, we showed that MEK inhibition downregulated PD-L1 expression and decreased the cell growth. In addition, our in vitro data support the ability of PD-L1 to regulate apoptosis in PTC. This insight into the role of PD-L1 in regulating apoptosis has been reported previously in a limited number of solid tumor cell lines [30,49,50,51].

## 4. Materials and Methods

### 4.1. Sample Selection

One thousand four-hundred and fifty-eight PTC patients diagnosed between 1989 and 2018 at King Faisal Specialist Hospital and Research Center (Riyadh, Saudi Arabia) with available archival tissue samples were included in the study. Clinico-pathological data were collected from case records, the details of which are summarized in Table 3. The Institutional Review Board of the hospital provided approval for the collection of archival samples. For this study, since only archival paraffin tissue blocks were used, a waiver of consent was obtained from the Research Advisory Council (RAC) on 06 February 2012 (RAC# 2110 031).

### 4.2. DNA Isolation

DNA was extracted from PTC formalin-fixed and paraffin-embedded (FFPE) tumor tissues utilizing a Gentra DNA isolation kit (Gentra, Minneapolis, MN, USA) according to manufacturer’s protocols, as elaborated in a previous study [52].

### 4.3. Sanger Sequencing Analysis

Sequencing of entire coding and splicing regions of exon 15 in the *BRAF* gene from 1257 PTC samples was carried out using Sanger sequencing technology. Primer 3 online software was utilized to design the primers (*BRAF*-EX15F1: AAACTCTTCATAATGCTTGCTCTG, *BRAF*-EX15R1: TTTCTAGTAACTCAGCAGCATCTCA). PCR and Sanger sequencing analyses were carried out as described previously [53]. A reference sequence was downloaded from NCBI GenBank. Sequencing results were compared with the reference sequence by Mutation Surveyor V4.04 (Soft Genetics, LLC, State College, PA, USA).

### 4.4. Tissue Microarray (TMA) Construction and Immunohistochemistry (IHC)

A tissue microarray (TMA) format was utilized for immunohistochemical analysis of the PTC samples. TMA was constructed as previously described [54]. Briefly, a modified semiautomatic robotic precision instrument (Beecher Instruments, Woodland, WI, USA) was used to punch tissue cylinders with a diameter of 0.6 mm from a representative tumor area of the donor tissue block and brought into the recipient paraffin block. Two 0.6 mm cores of PTC were arrayed from each case.

Tissue microarray slides were processed and stained manually as described previously [55]. Primary antibody against PD-L1 (E1L3N, 1:50 dilution, pH 9.0, Cell Signaling Technology, Danvers, MA, USA) was used. A membranous and/or cytoplasmic staining was observed. Only the membrane staining was considered for scoring. PD-L1 was scored as described previously [56]. Briefly, the proportion of positively stained cells was calculated as a percentage for each core and the scores were averaged across two tissue cores from the same tumor to yield a single percent staining score representing each cancer patient. For the purpose of statistical analysis, the scores were dichotomized. Cases showing an expression level of ≥ 5% were classified as over-expression and those with less than 5% as low expression. Only staining of tumor cells (not lymphocytes) was considered for percentage calculation.

### 4.5. Cell Culture

The PTC cell line BCPAP was obtained from Deutsche Sammlung von Mikroorganismen und Zellkulturen (DSMZ, Braunschweig, Germany), and TPC-1 was kindly provided by Dr. Bryan McIver (Department of Endocrinology, Mayo Clinic, Rochester, MN, USA). The K1 cell line was purchased from the American Type Culture Collection (ATCC, Manassas, VA, USA). Cell lines were cultured in RPMI 1640 media supplemented with 10% fetal bovine serum (FBS), 100 units/mL penicillin/streptomycin and 100 units/mL glutamine. These cell lines were authenticated in-house using short tandem repeat PCR and the results were in concordance with published data [57,58]. All experiments were performed using 5% FBS in RPMI 1640 media.

### 4.6. Reagents and Antibodies

BRAF inhibitor vemurafenib (PLX4032) and MEK inhibitor selumetinib (AZD6244) were purchased from Selleck Chemicals (Houston, TX, USA). PD-L1 antibody (ab205921) was obtained from Abcam (Cambridge, MA, USA). Antibodies against pMEK1/2 (9121), MEK1/2 (4694), pERK1/2 (4370), ERK1/2 (4695), AKT (9272), Bcl2 (2876), Bcl-XL (2764), cleaved caspase-3 (9661) and PARP (9542) were purchased from Cell Signaling Technology (Danvers, MA, USA). Antibodies against pAKT (sc-7985), caspase-3 (sc-56053) and GAPDH (sc-47724) were purchased from Santa Cruz Biotechnology, Inc. (Santa Cruz, CA, USA).

### 4.7. Clonogenic Assay

The PTC cell lines were seeded at a density of 500 cells per well in a 6-well plate. After attachment, fresh growth medium was added and cells were allowed to grow for 6–8 days. Cell colonies were fixed with formaldehyde (4%) and stained with crystal violet (2% in 10% methanol). The number of colonies in each well was counted and photographed.

### 4.8. Gene Silencing Using siRNA

PD-L1 siRNA and scrambled control siRNA were purchased from Ambion (Austin, TX, USA). Cells were transfected using Lipofectamine 2000 (Invitrogen, Carlsbad, CA, USA) for 6 h following which the lipid and siRNA complex was removed and fresh growth medium was added. After 48 h of transfection, cells were used for immuno-blotting.

### 4.9. Cell Lysis and Immuno-Blotting

Following treatment, PTC cells were lysed in phosphorylation lysis buffer containing 50 mM HEPES (pH 7.3), 150 mM NaCl, 1.5 mM MgCl_2_, 1.0 mM EDTA (pH 8.0), 100 mM NaF, 10 mM Na_2_H_2_P_2_O_7_, 200 µM Na_3_VO_4_ and 1X proteasome inhibitors (Roche pharmaceuticals, Basel, Switzerland). Lysed cells were spun at 14,000 rpm for 15 min at 4 °C and protein amounts were measured using a Bradford protein assay kit (Bio-Rad, Hercules, CA, USA). For immuno-blotting, equal amounts of protein (10 μg) were subjected to 10% SDS-PAGE gels, transferred to nitrocellulose membranes, blocked with 5% (*w*/*v*) non-fat dry milk in 1X TBST (25 mM Tris-HCl, pH 7.4, 137 mM NaCl, and 0.1% Tween 20) for 1 h and incubated with primary antibodies in 2% (*w*/*v*) non-fat dry milk in TBST for 1–2 h. The membranes were washed at least three times with TBST at 10 min intervals followed by a 1 h incubation with mouse or rabbit horseradish peroxidase-conjugated secondary antibody (1:5000). The membranes were developed with an enhanced chemiluminescence detection system according to the manufacturer’s instructions (ECL, Amersham, IL, USA). All uncropped western blot images are presented in Supplementary Materials Figure S1.

### 4.10. Statistical Analysis

The associations between clinico-pathological variables and protein expression were found using contingency table analysis and chi-square tests. A Mantel–Cox log-rank test was used to evaluate disease-free survival and recurrence-free survival. Survival curves were generated using the Kaplan–Meier method. A Cox proportional hazards regression model was used for multivariate analysis. Two-sided tests were used for statistical analyses with a limit of significance defined as *p* value < 0.05. Data analyses were performed using the JMP11.0 (SAS Institute, Inc., Cary, NC, USA) software package.

For all functional studies, data presented are means ± SD of three independent experiments, which were repeated at least two times with the same results. A Student’s *t*-test (two-tailed) was performed for statistical significance with *p* < 0.05 used as the cut-off.

## 5. Conclusions

This study suggests that PD-L1 could represent a useful prognostic marker for risk stratification in Middle Eastern PTC and that a PD-1/PD-L1 inhibitor could be a potential therapeutic option for aggressive PTC cancers, such as tall cell variant and *BRAF* mutation-positive patients that are unresponsive to standard PTC treatment. Furthermore, these data indicate that PTC tumor cells expressing PD-L1 may mediate intrinsic signaling and can affect survival beyond immune regulatory functions, which suggest a broader role for PD-L1 as a potential predictive marker for therapy response.

## Figures and Tables

**Table 1:** Association of clinico-pathological characteristics with PD-L1 expression in papillary thyroid carcinoma.

	Total		PD-L1 High			PD-L1 Low				*p* Value
	No.	%	No.	%		No.		%		
No. of Patients	1458		473	32.4		985		67.6		
Age (Years)										
<55	1189	81.5	389	32.7		800		67.3		0.6365
≥55	269	18.5	84	31.2		185		68.8		
Sex										
Female	1102	75.6	370	33.6		732		66.4		0.1014
Male	356	24.4	103	28.9		253		71.1		
Extrathyroidal Extension										
Absent	834	57.2	250	30.0		584		70.0		0.0203 (0.0271) *
Present	624	42.8	223	35.7		401		64.3		
pT										
pT1	400	28.5	130	32.5		270		67.5		0.5456
pT2	298	21.2	89	29.9		209		70.1		
pT3	595	42.4	204	34.3		391		65.7		
pT4	110	7.8	33	30.0		77		70.0		
pN										
pN0	582	44.4	176	30.2		406		69.8		0.0466 (0.0466) *
pN1	728	55.6	258	35.4		470		64.6		
pM										
pM0	1401	96.1	454	32.4		947		67.6		0.8836
pM1	57	3.9	19	33.3		38		66.7		
Stage										
I	1188	84.3	380	32.0		808		68.0		0.9322
II	152	10.8	53	34.9		99		65.1		
III	19	1.4	6	31.6		13		68.4		
IVA	19	1.4	5	26.3		14		73.7		
IVB	30	2.1	10	33.3		20		66.7		
Histology Type										
Classical Variant	955	65.5	334	35.0		621		65.0		<0.0001 (<0.0001) *
Follicular Variant	258	17.7	54	20.9		204		79.1		
Tall Cell Variant	135	9.3	54	40.0		81		60.0		
Other Variants	110	7.5	31	28.2		79		71.8		
*BRAF* Mutation										
Yes	707	56.2	250	35.4		457		64.6		0.0183 (0.0271) *
No	550	43.8	160	29.1		390		70.9		
Disease-Free Survival										
5 years			266	62.2	751		79.2		<0.0001	
Recurrence-Free Survival										
5 years			354	82.0	806		86.5		0.0006	

* p values in parentheses represent the Benjamini–Hochberg post hoc test *p* values. pT—pathologic tumor size; pN—pathologic lymphnode metastasis; pM—pathologic distant metastasis; PD-L1—Programmed Cell Death Ligand 1.

**Table 2:** Cox regression model analysis for prediction of disease-free survival and recurrence-free survival.

	Disease-Free Survival				Recurrence-Free Survival			
	Univariate		Multivariate		Univariate		Multivariate	
Clinico-pathological Variables	Risk Ratio (95% CI)	*p* Value	Risk Ratio (95% CI)	*p* Value	Risk Ratio (95% CI)	*p* Value	Risk Ratio (95% CI)	*p* Value
Age Above ≥55 years (vs. <55 years)	2.54 (2.03–3.16)	<0.0001 *	2.13 (1.59–2.87)	<0.0001 *	2.97 (2.31–3.80)	<0.0001 *	2.65 (1.90–3.70)	<0.0001 *
Sex Male (vs. Female)	0.59 (0.48–0.73)	<0.0001 *	0.67 (0.52–0.86)	0.0016 *	0.56 (0.44–0.71)	<0.0001 *	0.71 (0.53–0.95)	0.0224 *
Histology Tall Cell Variant (vs. Other Variants)	1.94 (1.41–2.61)	<0.0001 *	1.48 (1.04–2.05)	0.0236 *	1.27 (0.84–1.83)	0.2528	0.93 (0.59–1.40)	0.7422
Extrathyroidal Extension Present (vs. Absent)	2.27 (1.82–2.86)	<0.0001 *	1.40 (1.07–1.82)	0.0149 *	2.92 (2.24–3.85)	<0.0001 *	1.72 (1.25–2.36)	0.0008 *
Lymph Node Metastasis N1 (vs. N0)	2.33 (1.84–2.96)	<0.0001 *	1.72 (1.30–2.26)	0.0001 *	2.74 (2.09–3.63)	<0.0001 *	2.31 (1.65–3.23)	<0.0001 *
Distant Metastasis Present (vs. Absent)	3.99 (2.83–5.62)	<0.0001 *	2.29 (1.43–3.69)	0.0006 *	6.14 (4.25–8.62)	<0.0001 *	2.78 (1.68–4.60)	<0.0001 *
Stage IV (vs. I-III)	3.62 (2.39–5.27)	<0.0001 *	0.83 (0.46–1.49)	0.5305	6.25 (4.12–9.10)	<0.0001 *	0.81 (0.44–1.52)	0.5164
PD-L1 High (vs. Low)	2.04 (1.66–2.52)	<0.0001 *	2.08 (1.65–2.62)	<0.0001 *	1.51 (1.19–1.92)	0.0008 *	1.54 (1.18–2.00)	0.0013 *

* Significant *p* value.

**Table 3:** Clinico-pathological variables for the patient cohort (*n* = 1458).

Clinico-Pathological Variables	*n* (%)
Age	
Median	38.0
Range (IQR) ^	29.0–50.0
<55 years	1189 (81.5)
≥55 years	269 (18.5)
Gender	
Female	1102 (75.6)
Male	356 (24.4)
Histopathology	
Classical Variant	955 (65.5)
Follicular Variant	258 (17.7)
Tall Cell Variant	135 (9.3)
Others	110 (7.5)
Extrathyroidal Extension	
Absent	834 (57.2)
Present	624 (42.8)
pT	
T1	400 (27.4)
T2	298 (20.4)
T3	595 (40.9)
T4	110 (7.5)
Unknown	55 (3.8)
pN	
N0	582 (39.9)
N1	728 (49.9)
Nx	148 (10.2)
pM	
M0	1401 (96.1)
M1	57 (3.9)
Stage	
I	1188 (81.5)
II	152 (10.4)
III	19 (1.3)
IVA	19 (1.3)
IVB	30 (2.1)
Unknown	50 (3.4)
*BRAF* Mutation	
Present	707 (48.5)
Absent	550 (37.7)
Unknown	201 (13.8)
RAI Therapy	
Yes	1113 (76.3%)
No	345 (23.7)

^ IQR—Interquartile range; RAI—Radioactive iodine.

## Data Availability

The data presented in this study are available on request from the corresponding author.

## Supplementary Materials

The following are available online at https://www.mdpi.com/2072-6694/13/3/555/s1, Figure S1: Uncropped Western Blot Images.
