# Supplementary figures and images for: PD-L1 Is an Independent Prognostic Marker in Middle Eastern PTC and Its Expression Is Upregulated by BRAFV600E Mutation
Source: Cancers (Basel). 2021 Feb 1;13(3):555. doi: 10.3390/cancers13030555 (PMC7867170; doi:10.3390/cancers13030555)

# Figure S1. Uncropped Western Blot Images

## Figure 3A

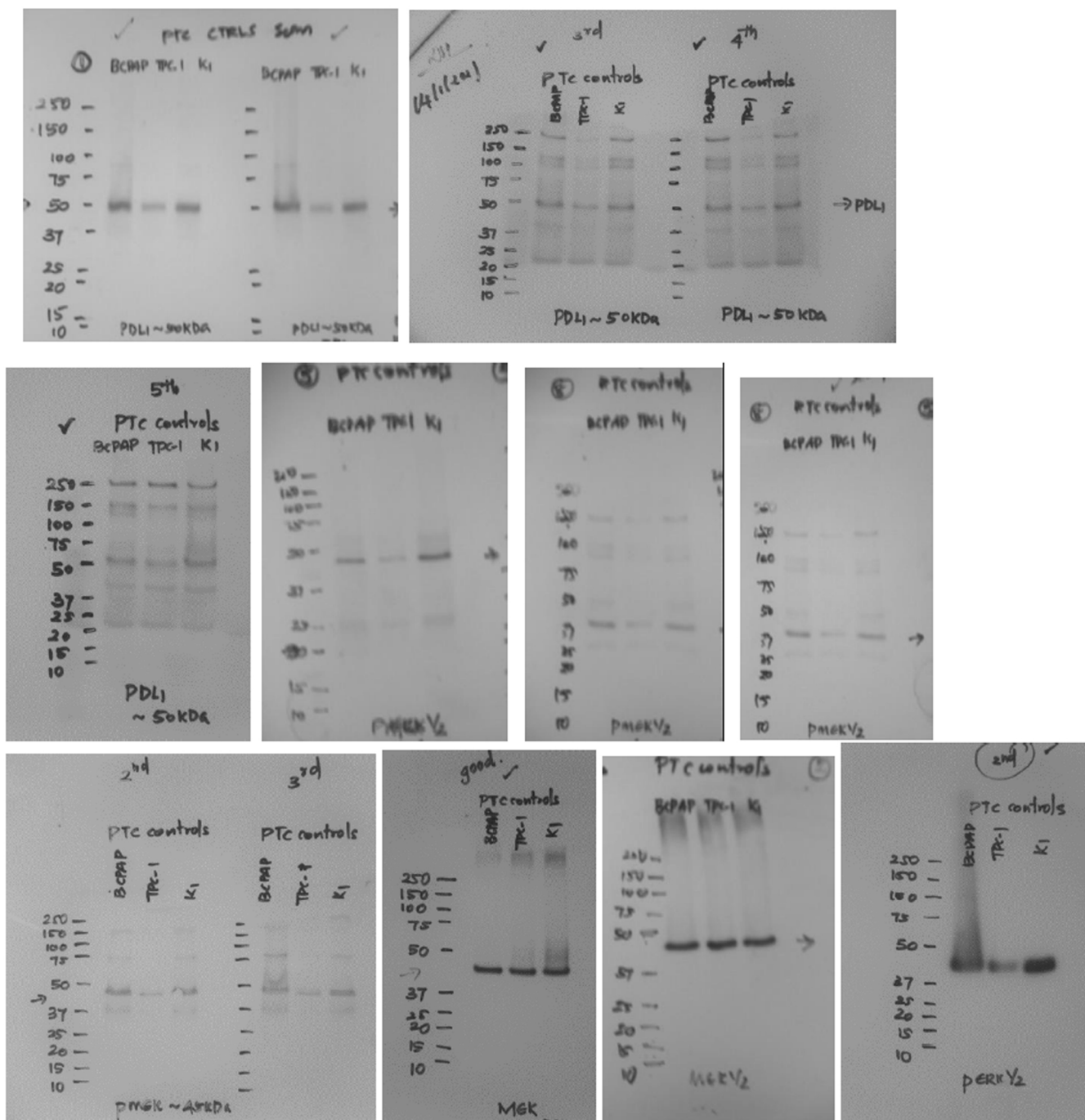

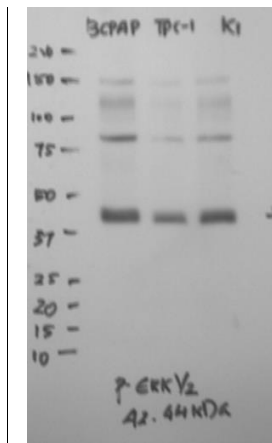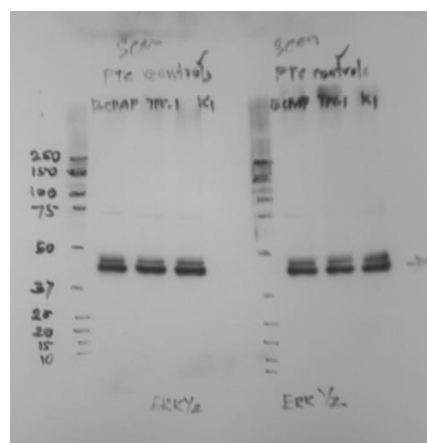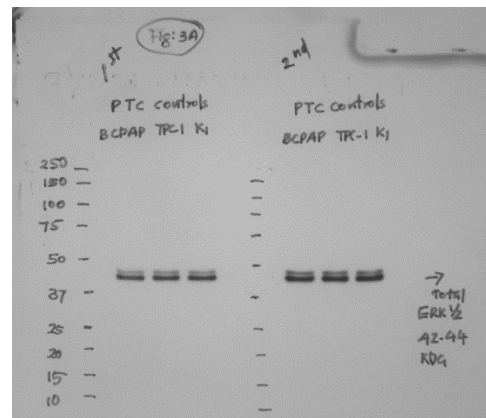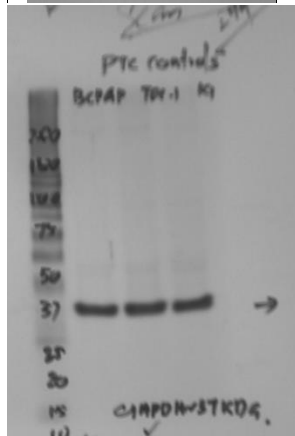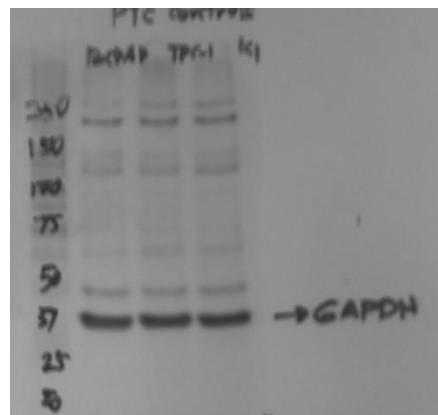

Figure 3C

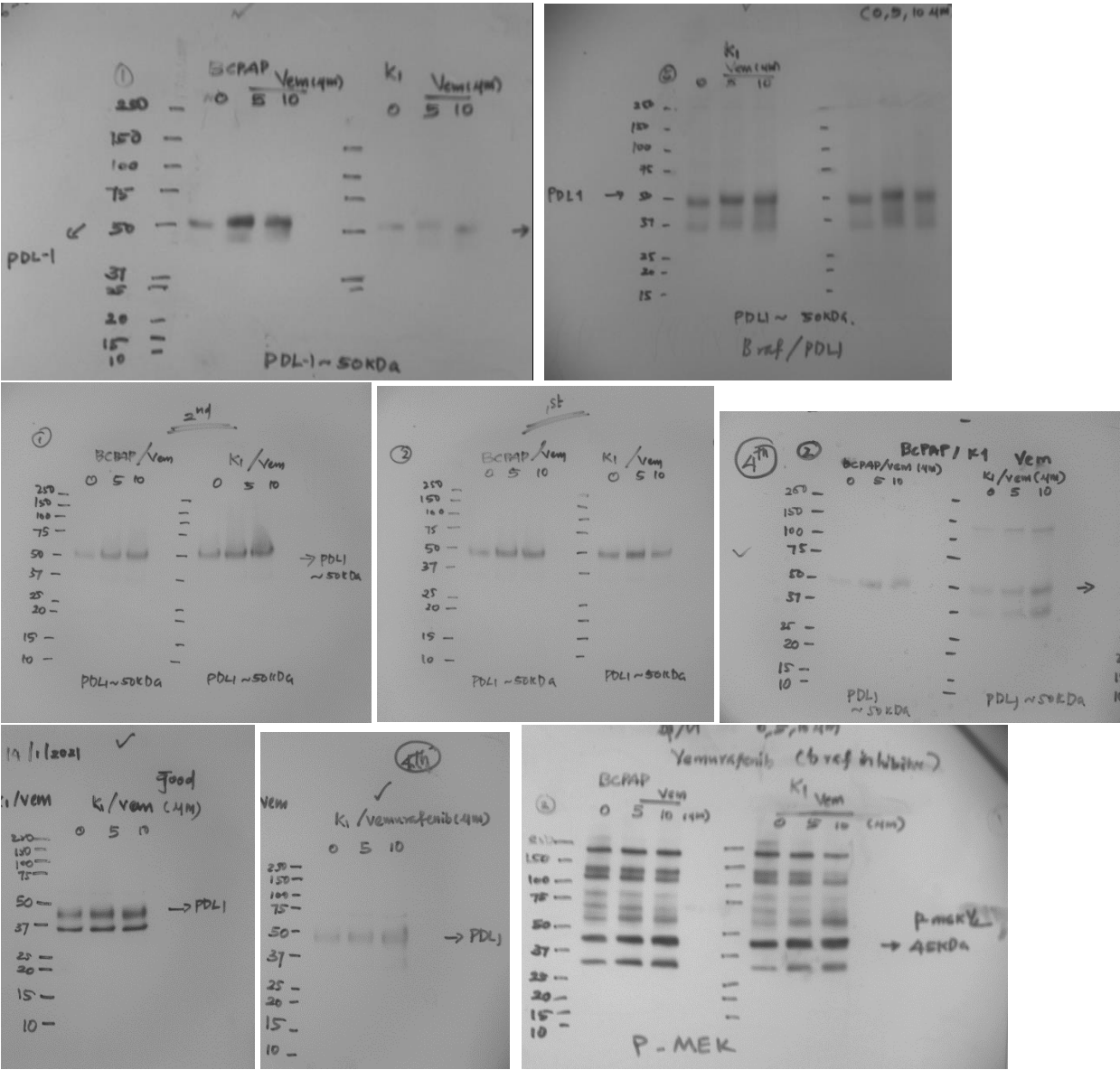

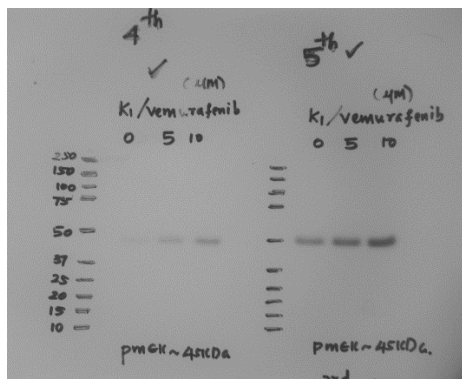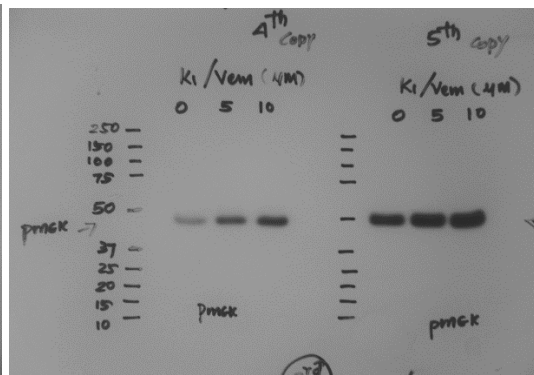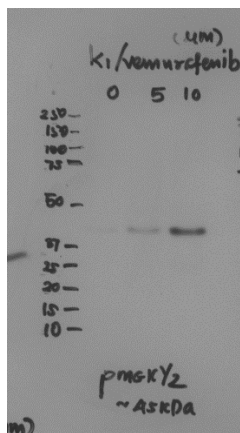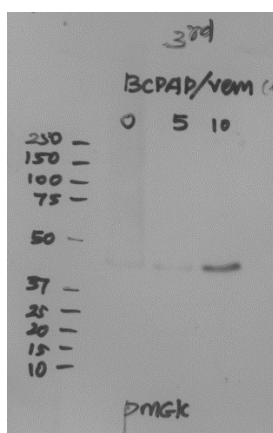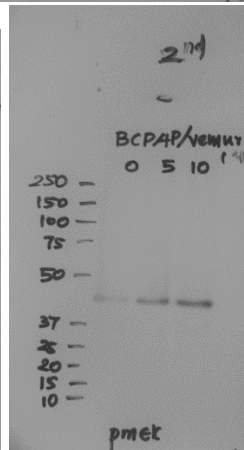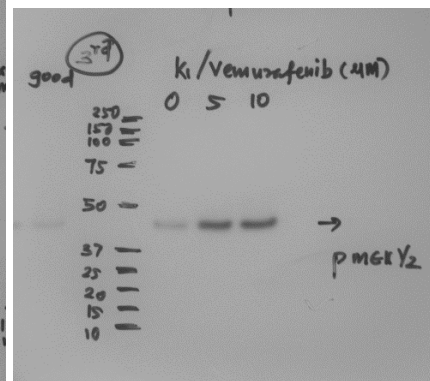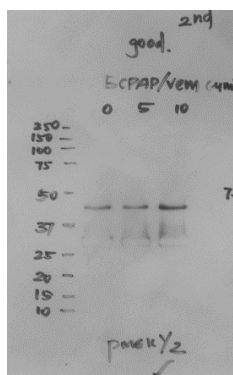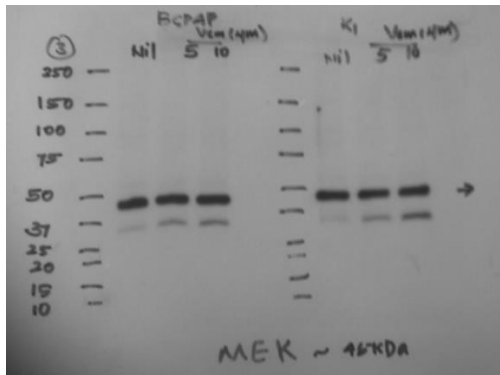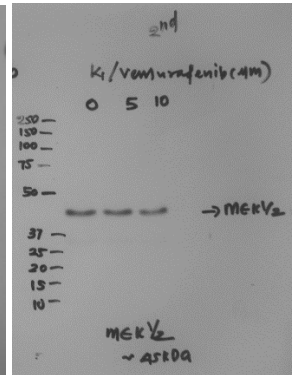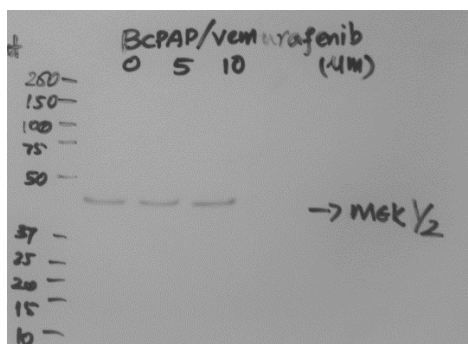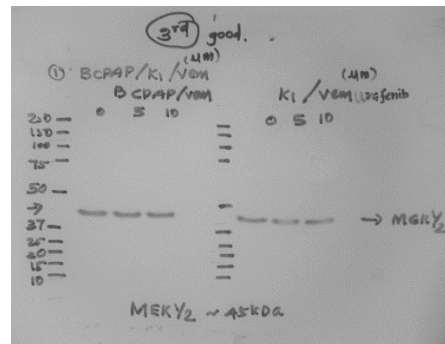

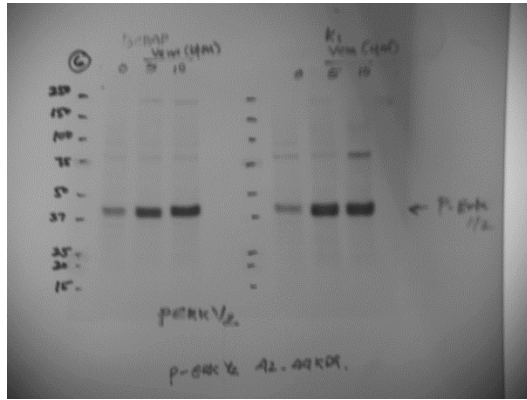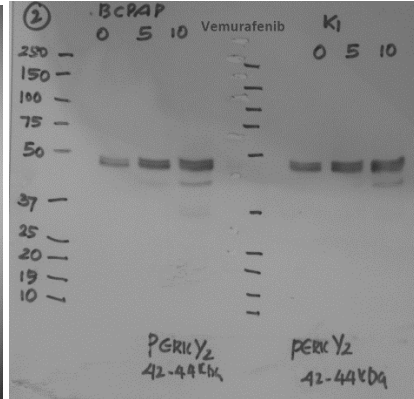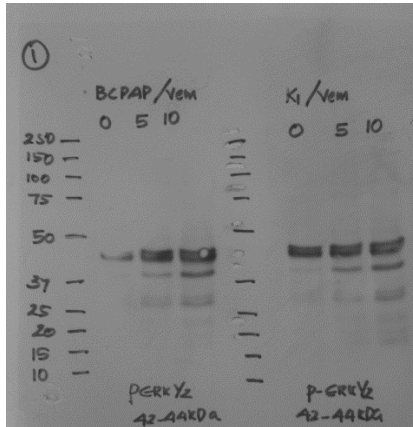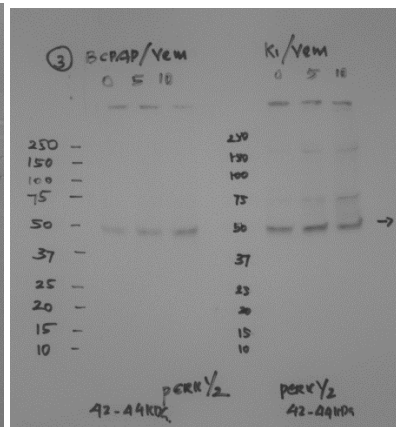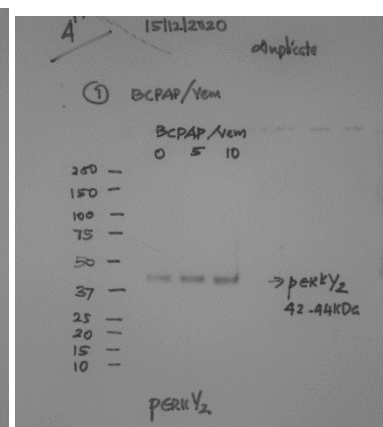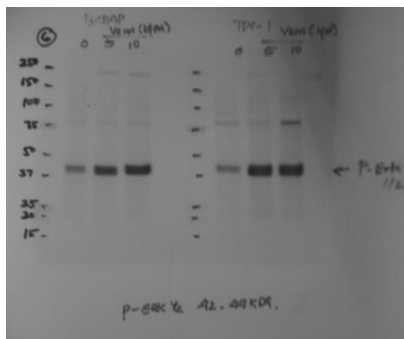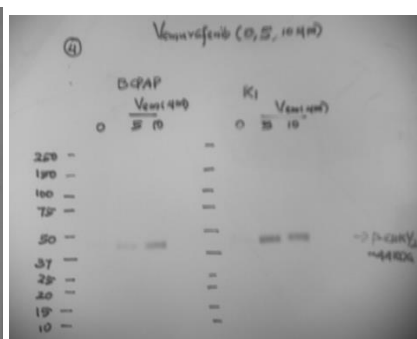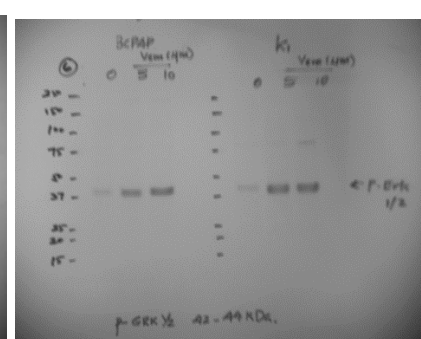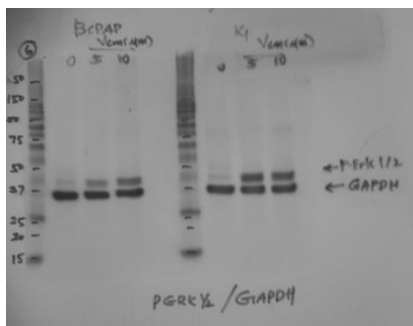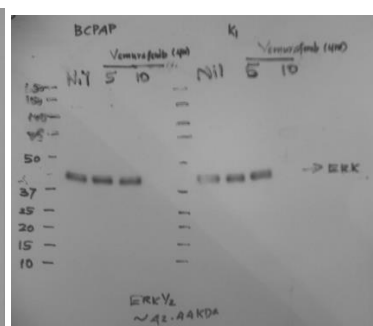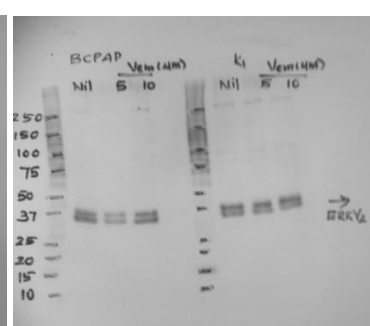

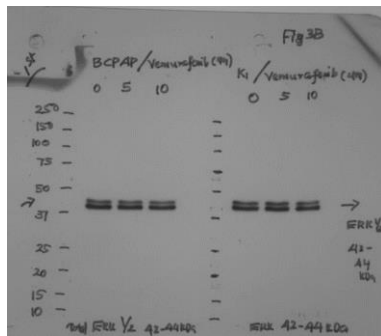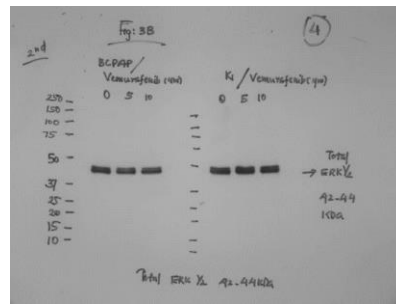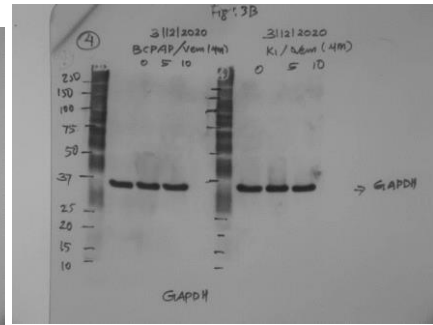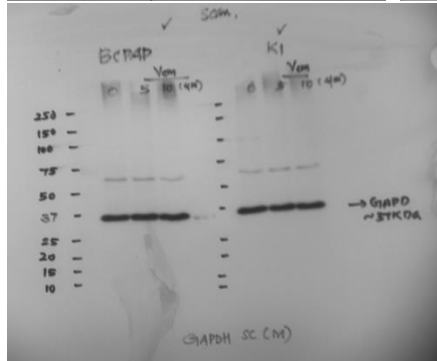

Figure 4A

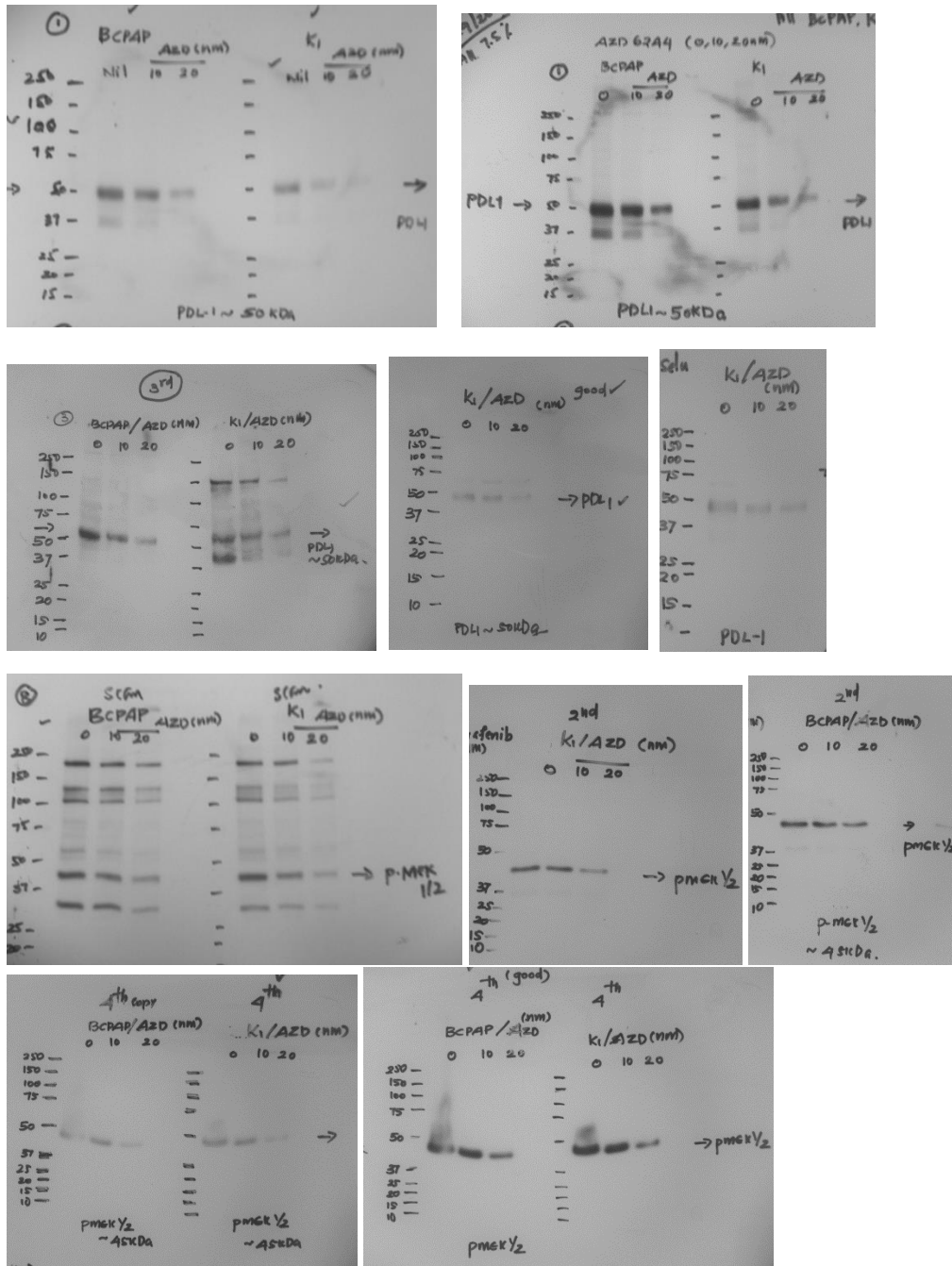

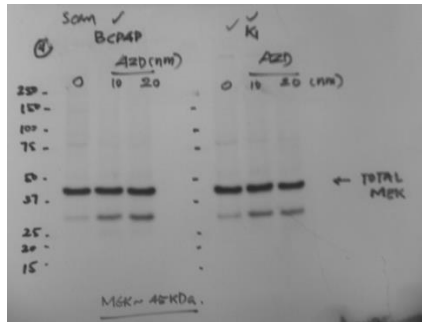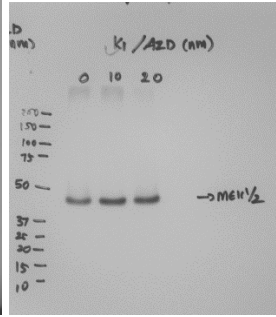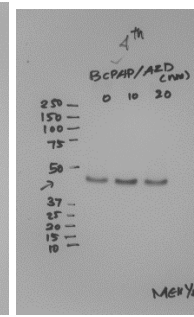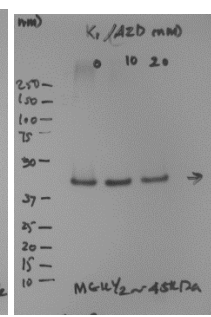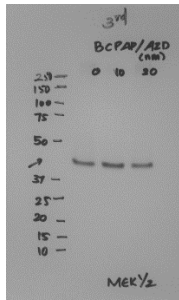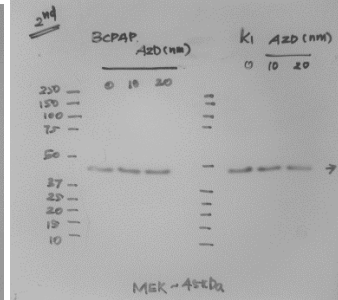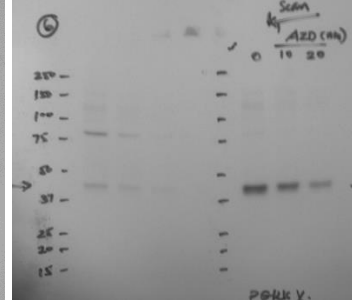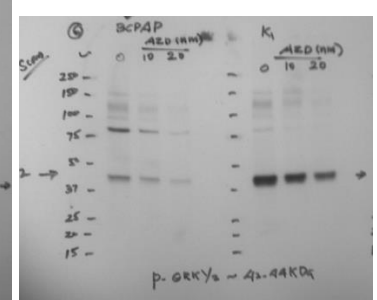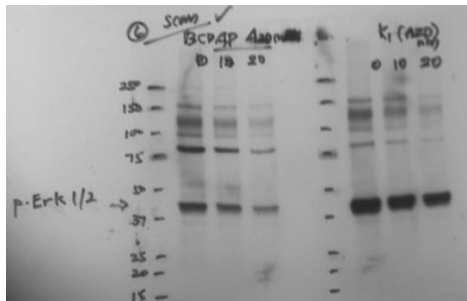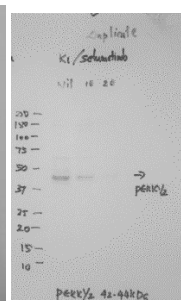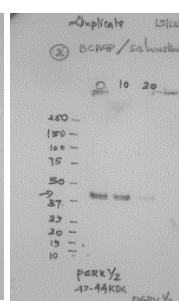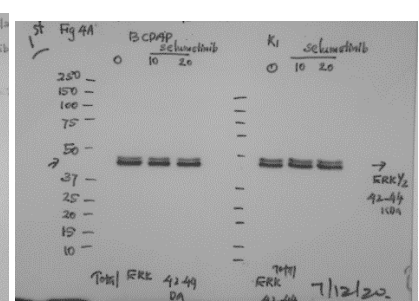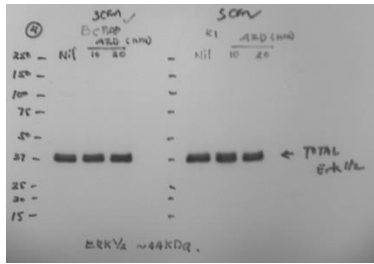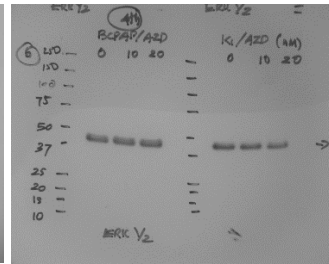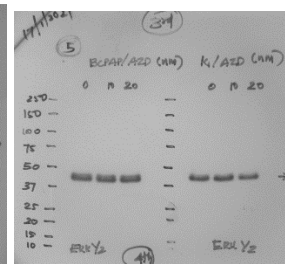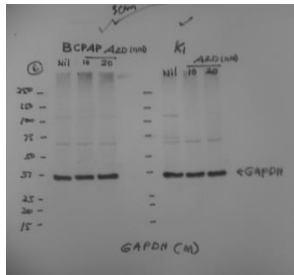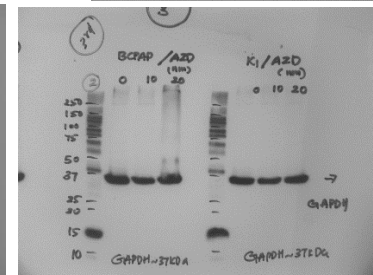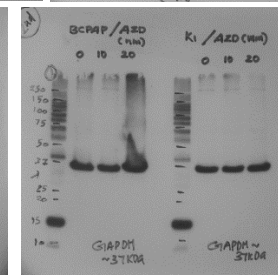

Figure 5C and E

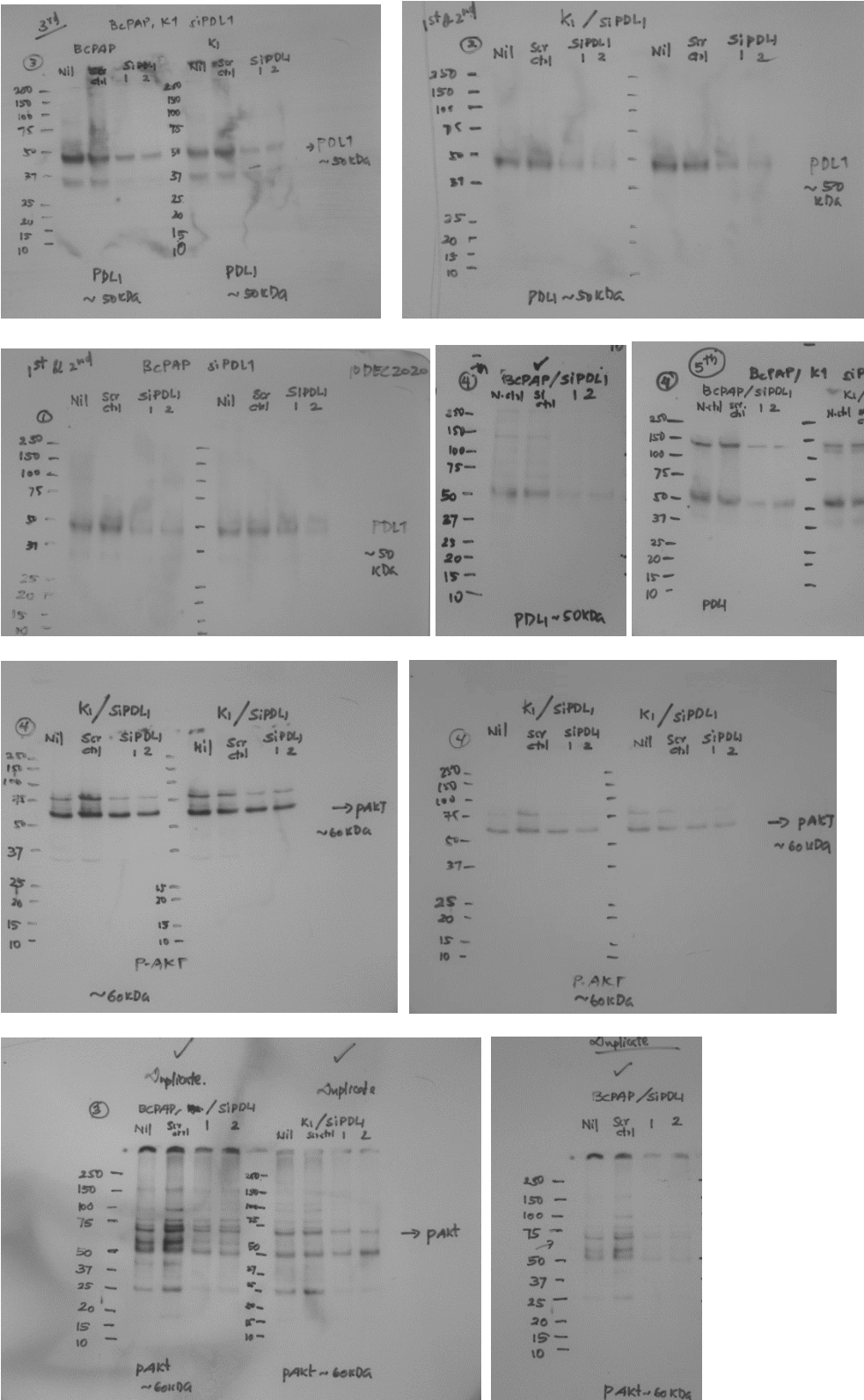

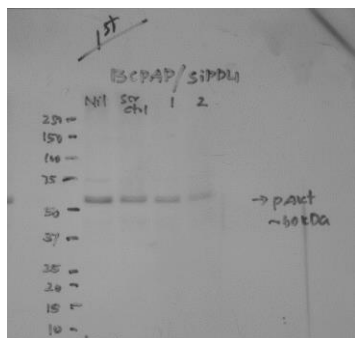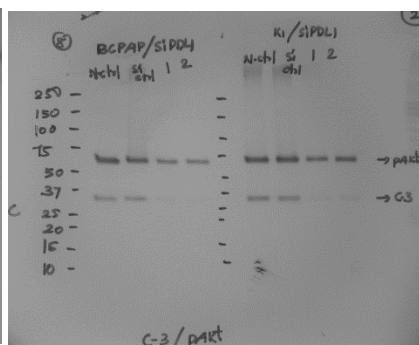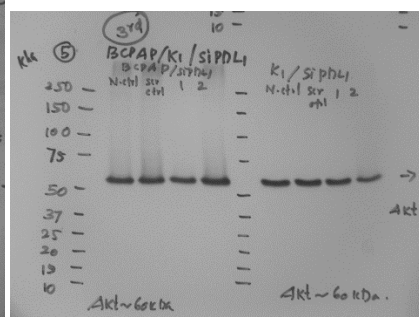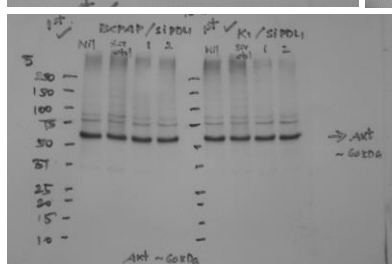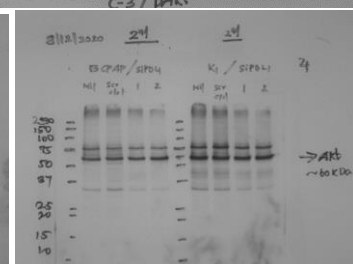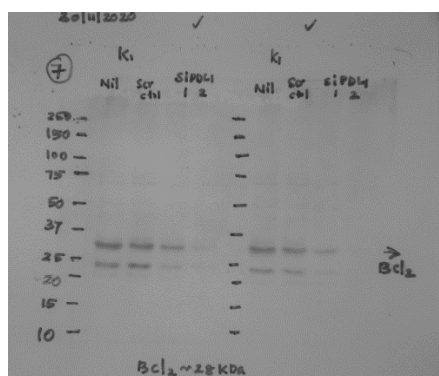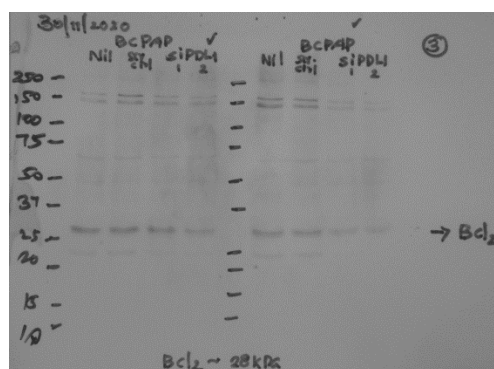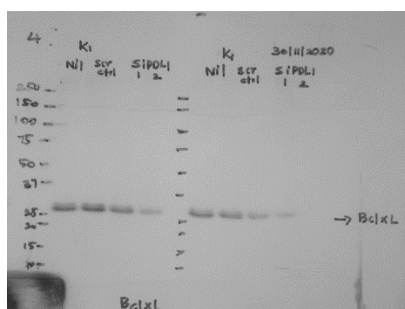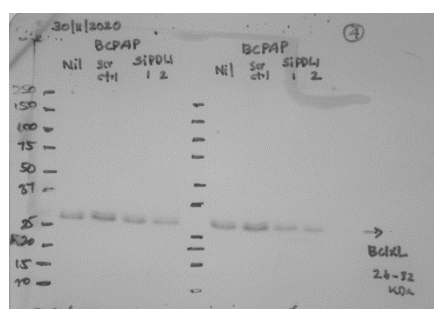

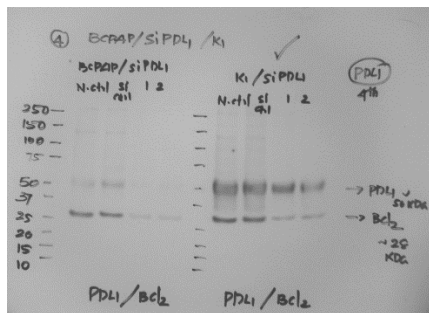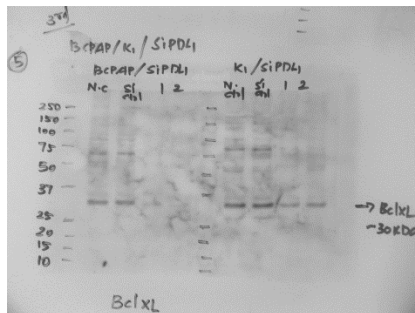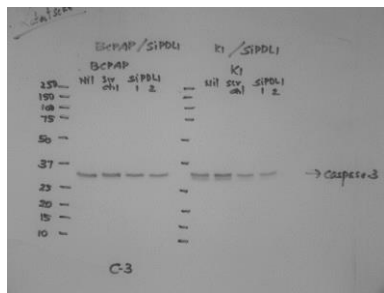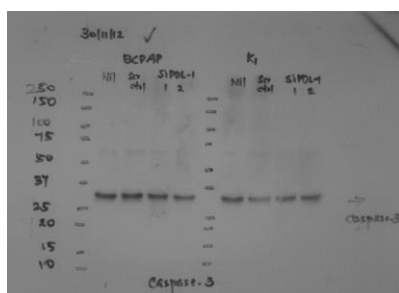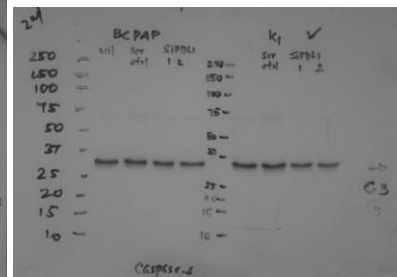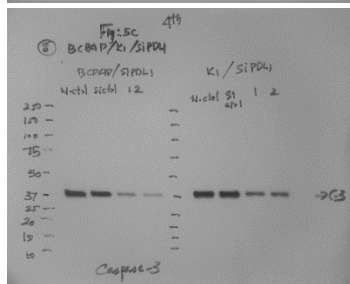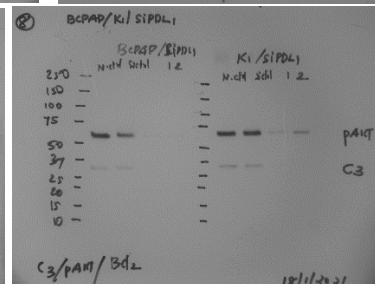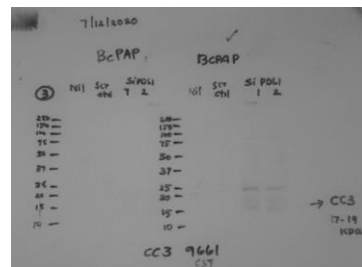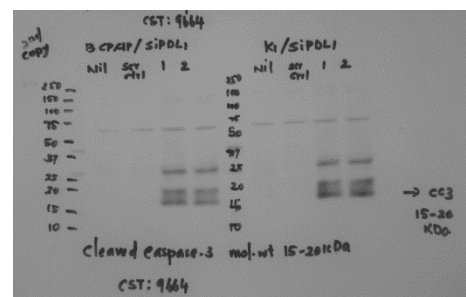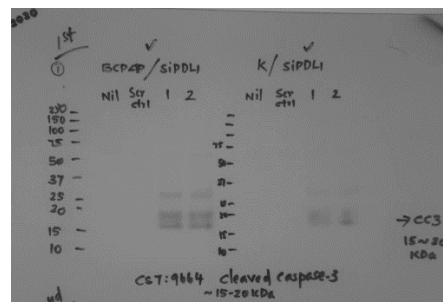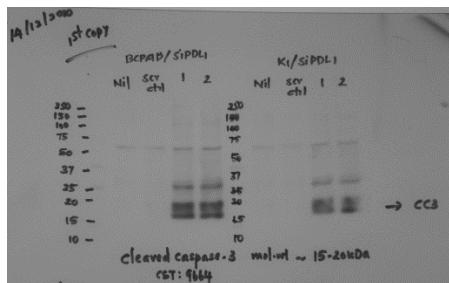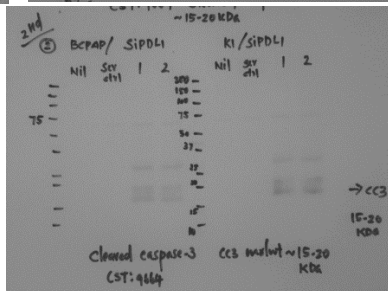

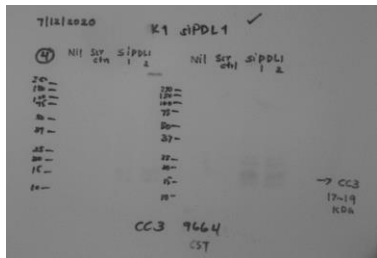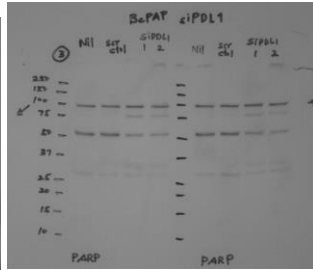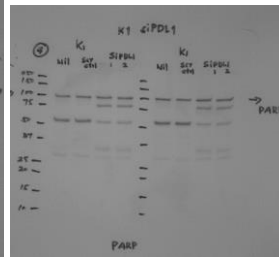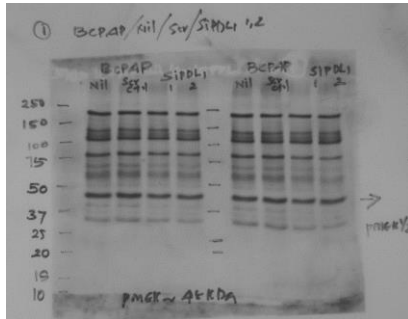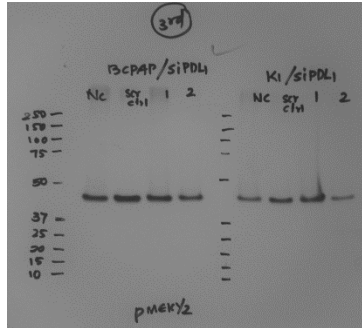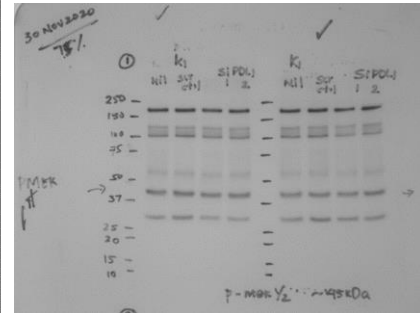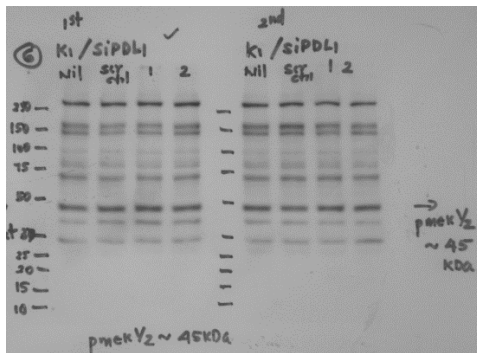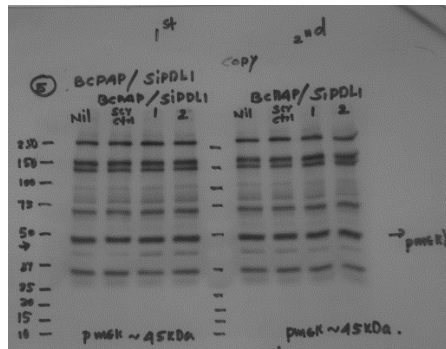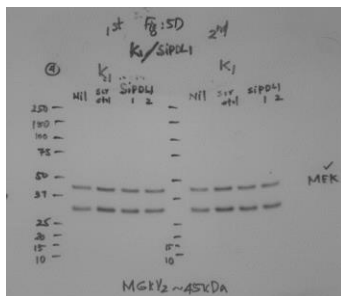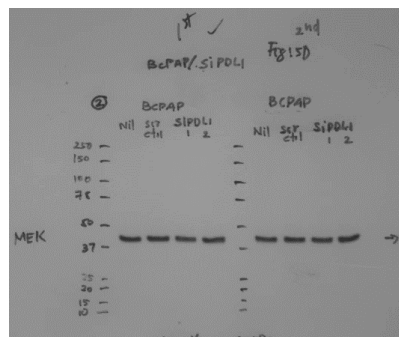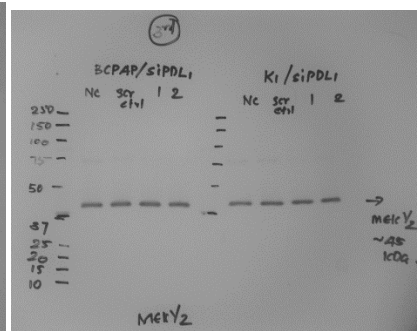

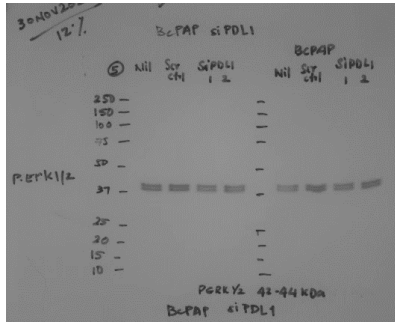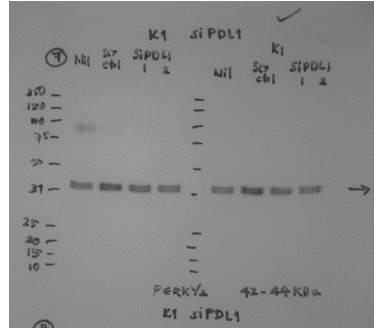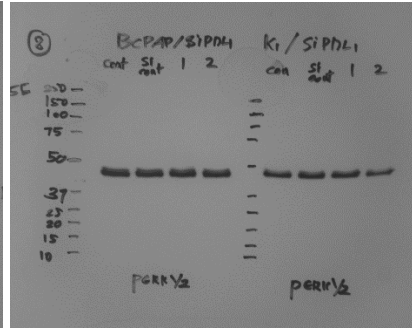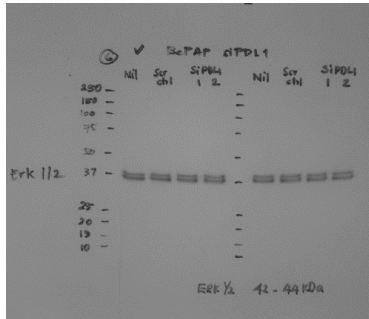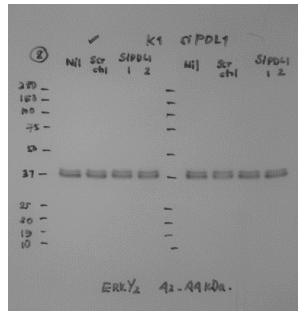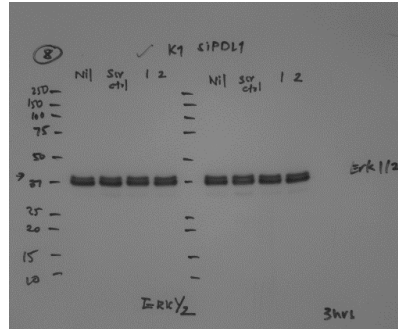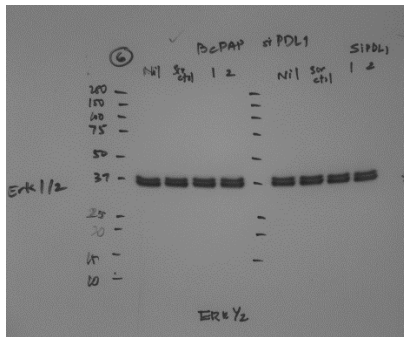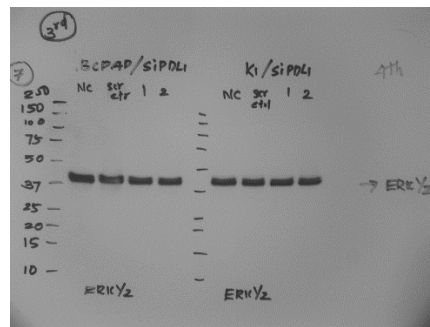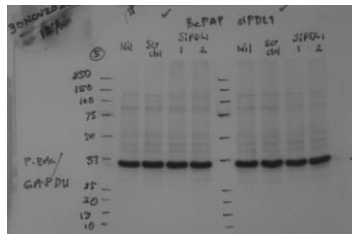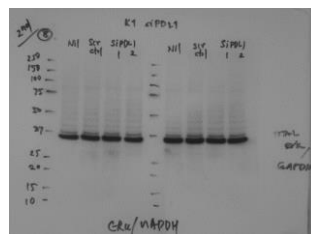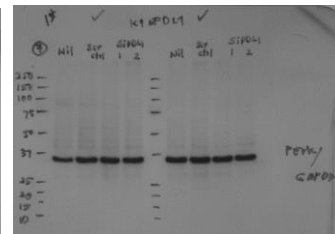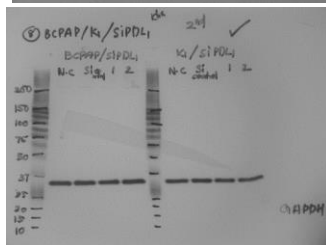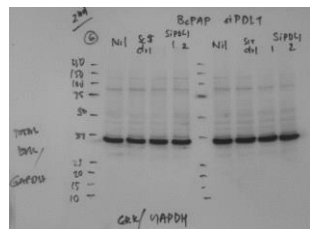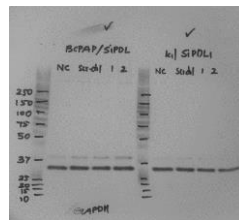

Supplement: Supplementary file 1 [file cancers-13-00555-s001.pdf]
